# Understanding Informed Consent: A Cross-Sectional Study of Objective and Self-Perceived Comprehension in Romania

**DOI:** 10.3390/healthcare14121777

**Published:** 2026-06-19

**Authors:** Alina Doina Tănase, Raluca Mioara Cosoroabă, Alexandra-Denisa Semenescu, Ioana Cristina Talpos-Niculescu, Daliana Emanuela Bojoga, Adriana Padure, Ștefania Dinu

**Affiliations:** 1 Department of Professional Legislation in Dental Medicine, Faculty of Dental Medicine, “Victor Babes” University of Medicine and Pharmacy, Eftimie Murgu Square No. 2, 300041 Timisoara, Romania; tanase.alina@umft.ro (A.D.T.); cosoroaba.raluca@umft.ro (R.M.C.); 2 Research Centre in Dental Medicine Using Conventional and Alternative Technologies, Faculty of Dental Medicine, “Victor Babes” University of Medicine and Pharmacy, Eftimie Murgu Square 2, 300041 Timisoara, Romania; 3 Department of Toxicology, Drug Industry, Management, Marketing and Dermatopharmacy, Faculty of Pharmacy, “Victor Babeș” University of Medicine and Pharmacy, 300041 Timisoara, Romania; 4 Research Centre for Pharmaco-Toxicological Evaluation, “Victor Babeș” University of Medicine and Pharmacy, 300041 Timisoara, Romania; 5 Department of Oro-Dental Diagnosis and Ergonomics, Faculty of Dental Medicine, “Victor Babes” University of Medicine and Pharmacy, Eftimie Murgu Square No. 2, 300041 Timisoara, Romania; adriana.padure@umft.ro; 6 University Clinic of Oral Rehabilitation and Dental Emergencies, Faculty of Dentistry, “Victor Babes” University of Medicine and Pharmacy Timisoara, Eftimie Murgu Square No. 2, 300041 Timisoara, Romania; mocuta.daliana@umft.ro; 7 Interdisciplinary Research Center for Dental Medical Research, Lasers and Innovative Technologies, Revolutiei 1989 Avenue No. 9, 300070 Timisoara, Romania; 8 Pediatric Dentistry Research Center (Pedo-Research), Department of Pediatric Dentistry, Faculty of Dental Medicine, “Victor Babes” University of Medicine and Pharmacy, Eftimie Murgu Square 2, 300041 Timisoara, Romania; dinu.stefania@umft.ro

**Keywords:** informed consent, patient comprehension, health literacy, decision-making

## Abstract

**Background/Objectives**: Informed consent (IC) is an essential component of medical practice; however, patients’ understanding of medical information remains challenging. This study aimed to assess both objective and self-perceived comprehension of information presented in an IC scenario and to identify factors associated with understanding. **Methods**: A cross-sectional study was conducted using an anonymous online questionnaire with 275 adult participants in Romania. The questionnaire included a standardized IC scenario followed by comprehension assessment questions. Each correct answer was assigned one point, generating a total comprehension score ranging from 0 to 8. Self-perceived comprehension was evaluated using a Likert scale. Statistical analyses included descriptive statistics to summarize participant characteristics and questionnaire responses, Spearman’s correlations to examine associations between self-perceived comprehension and objective comprehension scores, independent samples *t*-tests and ANOVA to compare comprehension scores across participant groups, and multiple linear regression to identify independent predictors of comprehension. **Results**: The mean comprehension score was 6.81 ± 1.48, indicating a generally high level of understanding. A moderate positive correlation was observed between objective and self-perceived comprehension (*ρ* = 0.35, *p* < 0.001). Non-healthcare participants achieved slightly higher scores than healthcare field participants (*p* = 0.046), while educational level was not significantly associated with comprehension score (*p* = 0.566). Multiple linear regression analysis identified self-perceived comprehension as a significant independent predictor of the comprehension score (*β* = 0.381, *p* < 0.001). **Conclusions**: Although the overall level of comprehension was high, discrepancies between self-perceived comprehension and objective comprehension were identified. These findings highlight the importance of patient-centered communication strategies and the need to actively verify patient understanding during the informed consent process to support truly informed decision-making.

## 1. Introduction

Informed consent (IC) is an essential component of modern medical practice and a fundamental ethical principle that reflects respect for patient autonomy. It is a process by which patients receive relevant information about a proposed medical intervention, including its purpose, potential benefits and risks, and available alternatives, to support informed decision-making [1,2]. For IC to be valid, patients must have the capacity to consent, act voluntarily, and retain the right to accept or refuse the proposed medical procedure [3].

Despite its importance, patients’ understanding of the information provided during the IC process remains limited. Evidence from the literature indicates that patients often recall only a small proportion of the information discussed and that their understanding is frequently overestimated. Key components, particularly potential risks and alternatives, are often poorly understood. In addition, informed consent documents are often written in complex medical terminology, which may be difficult for many patients to interpret [2].

Health literacy is a key factor in understanding biomedical information. It refers to the capacity to access, understand, and use health-related information to make appropriate decisions. Low levels of health literacy are associated with difficulty interpreting medical information. However, comprehension during the IC process is influenced by multiple interrelated factors. In addition to health literacy, demographic characteristics such as age and educational level, communication-related factors such as language clarity, previous healthcare experience, and emotional factors including anxiety may all affect patients’ understanding of medical information [4,5,6].

To improve comprehension, IC should be presented in clear, accessible language. Ineffective communication may lead patients to consent without fully understanding the information. Therefore, effective communication between healthcare professionals and patients is essential for clarifying aspects of medical interventions and supporting truly informed decisions [7,8,9].

In recent years, attention has increasingly focused on strategies to simplify and improve the IC process, including the use of adapted language and alternative information delivery methods, such as audiovisual tools [10,11,12]. Although these approaches have been shown to improve understanding, discrepancies between patients’ self-perceived comprehension and objective comprehension persist [7,13].

Based on previous evidence, we hypothesized that (1) objective comprehension would be positively associated with self-perceived comprehension and (2) participant characteristics, including educational level and healthcare background, would be associated with objective comprehension.

Thus, there remains a need to better understand the relationship between objective comprehension and self-perceived comprehension in the context of informed consent.

Therefore, the aim of this study was to evaluate adults’ understanding of information presented in a standardized informed consent scenario. The study also examined the relationship between objective and self-perceived comprehension and explored factors associated with comprehension.

## 2. Materials and Methods

### 2.1. Study Design

This cross-sectional observational study was designed to assess how Romanian adults understand information presented in a standardized IC scenario. The study was conducted by providing an anonymous online questionnaire to individuals aged 18 years or older.

Data collection took place between February 2026 and May 2026. Participation was voluntary, and no personally identifiable data were collected. All participants were informed about the purpose of the study and provided IC before completing the questionnaire.

The study protocol was approved by the Ethics Committee of the ‘Victor Babeș’ University of Medicine and Pharmacy, Timișoara (No. 45/26 January 2026), in compliance with the Declaration of Helsinki.

### 2.2. Participants

Participants were recruited using a convenience sampling approach through online distribution of the questionnaire. The questionnaire was created using Google Forms and disseminated via Facebook, WhatsApp, and personal e-mail networks. The questionnaire was primarily distributed to individuals from the general population, most of whom did not have a healthcare background.

No a priori sample size calculation was performed. Given the exploratory nature of the study, recruitment was based on voluntary participation during the study period. All eligible responses received during the recruitment period were considered for inclusion. To improve response quality, an attention-check item was included, and responses failing this check were excluded from the analysis. Eligibility criteria included age ≥18 years and completion of the questionnaire with consent to participate.

**Figure 1:**
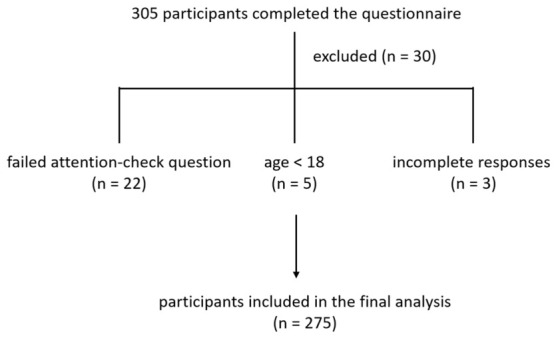
Flowchart of participant selection, exclusions, and inclusion in the final analysis.

A final sample of 275 participants was included in the analysis. Details of participant selection, exclusions, and reasons for exclusion are presented in Figure 1.

### 2.3. Data Collection

Data were collected using a structured online questionnaire specifically developed for this study. The questionnaire consisted of several sections:

1. Sociodemographic data: age, gender, area of residence, education level, professional status, and healthcare background. Additional questions assessed prior experience with IC and frequency of interaction with the healthcare system (Q1–Q8).
2. Self-reported familiarity with medical terminology and medical explanations: assessed using two Likert-scale items evaluating participants’ perceived understanding of medical terminology and clarity of medical explanations (Q9–Q10).
3. Standardized scenario: a description of a minimally invasive diagnostic medical procedure, including information on purpose, benefits, risks, alternatives, patient rights, and data confidentiality.
4. Objective comprehension assessment: eight multiple-choice questions (Q11–Q18), each with a single correct answer.
5. Self-perceived comprehension and attitudes: including self-perceived comprehension (Q19), need for additional explanations, and willingness to sign IC (Q20–Q21).
6. Evaluation of text clarity and length (Q22–Q23).
7. Attention-check item: included to ensure response validity.

The questionnaire was specifically developed for this study based on the essential elements of IC described in medical ethics. The comprehension section consisted of eight objective questions assessing participants’ understanding of key IC components, including the purpose of the procedure, potential benefits and risks, alternatives, confidentiality, voluntary participation, and the consequences of refusal. Each question was assigned equal weight and contributed one point to the total comprehension score. Although the items addressed different aspects of IC, all were considered essential components of IC understanding and were therefore scored equally.

To establish face and content validity, the questionnaire was reviewed by a multidisciplinary panel of researchers, physicians, and university faculty members with expertise in medicine and research methodology. Feedback regarding clarity, relevance, and comprehensibility was incorporated before final administration.

A pilot test involving eight participants was conducted to evaluate question clarity, wording, and completion time. Minor linguistic adjustments were made following pilot testing, while the overall structure and content of the questionnaire remained unchanged.

Formal assessment of reliability, item difficulty, and readability was not performed. However, clarity, comprehensibility, wording, and completion time were evaluated during expert review and pilot testing, and minor revisions were implemented accordingly.

The questionnaire was originally developed in Romanian and subsequently translated into English for publication purposes. The full version is available as Supplementary Material.

### 2.4. Statistical Analysis

Statistical analyses were performed using JASP software (version 0.18.3; JASP Team, University of Amsterdam, Amsterdam, The Netherlands; https://jasp-stats.org).

Descriptive statistics were used to summarize the data. Continuous variables were reported as means and standard deviations (SD), while categorical variables were presented as frequencies and percentages.

To assess comprehension, each correct response to questions Q11–Q18 was assigned one point, resulting in a total score ranging from 0 to 8. Higher scores indicated better understanding. The total score was calculated by summing the number of correct responses.

The proportion of correct answers for each item was expressed as a percentage.

Likert-scale responses were treated as ordinal variables. Associations between continuous and ordinal variables were assessed using Spearman’s correlation coefficient.

Independent-samples *t*-tests were used to compare comprehension scores according to healthcare background (healthcare vs. non-healthcare), educational level (university vs. non-university), and area of residence (urban vs. rural). One-way ANOVA was used to compare comprehension scores across categories of willingness to sign the IC form (yes, no, depends on the situation).

A multiple linear regression model was constructed to identify independent predictors of the comprehension score. Multivariable regression was used to adjust for potential confounding by the variables included in the model. Predictor variables were selected a priori based on their theoretical relevance to IC comprehension and their availability within the collected dataset. The variables included in the model were self-perceived comprehension level (Q19), healthcare background (yes/no), and education level (university vs. non-university). Other potentially relevant factors, such as age, sex, prior experience with informed consent, frequency of healthcare interaction, and health literacy, were not included in the final model and may also influence comprehension. Regression coefficients (*β*) and corresponding *p*-values were reported.

Prior to conducting the regression analysis, key assumptions of linear regression were assessed. Linearity, homoscedasticity, and normality of residuals were evaluated through visual inspection of residual-versus-predicted value plots and Q–Q plots. Multicollinearity was assessed using variance inflation factor (VIF) values and tolerance statistics. All VIF values ranged from 1.003 to 1.009, indicating no evidence of problematic multicollinearity.

A *p*-value < 0.05 was considered statistically significant.

A post hoc power analysis was performed for the final multiple linear regression model. Based on the observed effect size (*R^2^* = 0.16), a sample size of 275 participants, three predictors, and an alpha level of 0.05, the achieved statistical power exceeded 99%.

## 3. Results

### 3.1. Participant Characteristics

A total of 305 participants completed the questionnaire. After excluding participants who failed the attention test (*n* = 22), those under 18 years of age (*n* = 5), and incomplete responses (*n* = 3), 275 participants were included in the final analysis.

The mean age of the participants was 32.0 ± 13.2 years (range: 18–71). The majority of respondents were female (69.8%), while 30.2% were male. Most participants came from urban areas (81.8%), compared with a smaller percentage from rural areas (18.2%).

In terms of educational attainment, over half of the participants had university degrees (51.6%), followed by postgraduate degrees (19.3%) and high school degrees (26.5%). Lower levels of education were poorly represented.

The study sample was predominantly composed of individuals without a healthcare background, with only 8.4% reporting work in the healthcare field.

Regarding professional status, the majority of respondents were employed (57.5%), followed by students (33.1%), with other categories representing a small proportion. Table 1 displays all socio-demographic information.

### 3.2. Objective Comprehension of the Informed Consent Scenario

The mean comprehension score was 6.81 ± 1.48.

**Figure 2:**
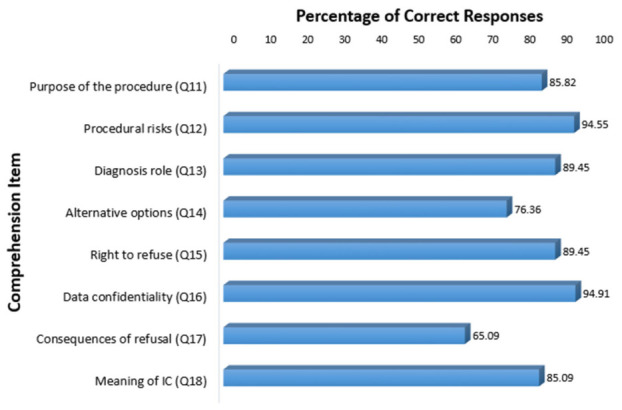
Percentage of correct responses for each objective comprehension item (Q11–Q18).

The proportion of correct answers varied across items. The highest rates of correct answers were recorded for questions Q16 (94.91%) and Q12 (94.55%), followed by Q13 (89.45%) and Q15 (89.45%). Moderate levels of correct answers were observed for Q11 (85.82%) and Q18 (85.09%). Conversely, lower rates were identified for Q14 (76.36%), and the lowest proportion was recorded for Q17 (65.09%). These data are presented in Figure 2.

Regarding the need for additional explanations, the majority of participants stated that they would not need additional explanations (52%), while 31.27% indicated that they would need additional clarification, and 16.73% were not sure.

### 3.3. Associations Between Objective Comprehension and Participant Characteristics

Associations between objective comprehension scores and participant characteristics were examined using correlation analyses and group comparisons.

The mean self-perceived comprehension score was 4.22 out of 5, suggesting that participants generally perceived themselves as understanding the information presented.

In terms of willingness to sign IC, 56% of participants reported they would be willing to sign, 38.18% indicated their decision would depend on the situation, and a smaller proportion (5.82%) stated they would not sign.

Additionally, a moderate positive correlation was observed between the objective comprehension score and self-perceived comprehension (*ρ* = 0.35, *p* < 0.001), showing that higher self-perceived comprehension was associated with higher scores.

No significant correlation was observed between self-reported understanding of medical terminology and comprehension score (*ρ* = 0.062, *p* = 0.305).

Also, no significant differences in comprehension scores were observed according to participants’ willingness to sign IC (*F* = 0.724, *p* = 0.486), with a negligible effect size (*η^2^* = 0.005).

Regarding the area of residence, no significant differences in comprehension scores were observed between participants from urban and rural areas (*t* = −1.307, *p* = 0.192), although the average score was slightly higher in urban areas.

A significant difference was found between healthcare and non-healthcare participants in comprehension scores (*t* = 2.007, *p* = 0.046), with a small-to-moderate effect size (Cohen’s *d* = 0.437). Non-healthcare participants had a slightly higher mean score (6.86 ± 1.43) compared to healthcare participants (6.22 ± 1.93).

No statistically significant difference was observed between participants with and without university education in terms of comprehension score (*t* = 0.575, *p* = 0.566). The mean scores were similar between the two groups (6.89 ± 1.56 vs. 6.77 ± 1.45).

### 3.4. Predictors of Objective Comprehension

A multivariate linear regression analysis was conducted to identify independent predictors of the comprehension score while adjusting for potential confounders. The model was statistically significant (*F* = 17.16, *p* < 0.001) and explained 16% of the variance in the score (*R^2^* = 0.16).

A post hoc power analysis indicated that the final regression model achieved a statistical power of >99% to detect the observed effect size.

Diagnostic analyses indicated no major violations of regression assumptions. Residual plots supported linearity and homoscedasticity, Q-Q plots suggested acceptable residual normality, and VIF values ranged from 1.003 to 1.009. Self-perceived comprehension level was a significant positive predictor of the score (*β* = 0.381, *p* < 0.001).

The health profession was also significantly associated with comprehension scores, with health profession participants showing slightly lower scores (*β* = −0.139, *p* = 0.014). Whereas education level was not a significant predictor (*β* = −0.027, *p* = 0.627) (Table 2).

## 4. Discussion

Over time, patients have reported that informed consent forms are often overly complex, which may hinder adequate understanding and highlight the need for additional measures to improve this process. Consequently, optimizing the IC process is essential to ensure clear communication and support informed decision-making [7,14]. Previous studies have demonstrated that simplifying both the structure and language of consent forms can significantly improve participants’ comprehension [13].

### 4.1. Overall Comprehension of the Informed Consent Scenario

The present study evaluated the level of understanding of information presented in a standardized IC scenario and explored the relationship between objective and self-perceived comprehension. Participants achieved a mean comprehension score of 6.81 out of 8. However, because no validated cut-off values are available for this study-specific instrument, the findings should be interpreted with caution. These results are consistent with existing literature emphasizing the importance of communication quality and health literacy in facilitating understanding of medical information [1,5]. For instance, Nishimura et al. demonstrated that simplified consent forms significantly improve patient understanding [15], while other studies have shown that combining clear explanations with adapted materials enhances comprehension [16].

A detailed analysis of individual items revealed variability in understanding depending on the type of information. Concrete aspects, such as the purpose of the procedure or associated risks, were better understood, whereas more abstract concepts, including the implications of IC and the consequences of refusal, were more difficult to interpret. These findings are in line with previous research indicating that patients often struggle with the ethical and legal dimensions of IC, even when information is provided in detail [3,17].

### 4.2. Objective Versus Self-Perceived Comprehension

The moderate correlation between self-perceived and objective comprehension, together with the lack of association between familiarity with medical terminology and actual comprehension, highlights a discrepancy between subjective perception and real understanding. This finding aligns with previous literature suggesting that patients’ perception of being well-informed does not necessarily reflect their actual level of comprehension. A systematic review reported that adequate understanding of IC is achieved in fewer than one-third of studies, despite patients frequently perceiving themselves as sufficiently informed [18].

Furthermore, our study found no significant correlation between self-reported medical knowledge and objective comprehension scores. This suggests that patients often overestimate their ability to understand complex medical documents. This “illusion of knowing” means that subjective confidence is not a reliable indicator of whether a patient truly understands the information provided [19].

### 4.3. Factors Associated with Comprehension

An interesting and somewhat counterintuitive finding was the significantly higher comprehension score observed among non-healthcare participants compared to those with a healthcare background. However, this result should be interpreted with caution due to the small number of healthcare participants (*n* = 23) relative to non-healthcare participants (*n* = 252). Although the difference reached statistical significance, it may have been influenced by sampling imbalance and should therefore be considered exploratory. Previous research has suggested that professional familiarity with medical information may influence information processing [20]; however, the present study was not designed to investigate potential underlying mechanisms. Further studies involving larger and more balanced samples are needed to determine whether this finding can be replicated.

Health literacy is widely recognized as an important factor in the IC process, as it is associated with differences in the ability to understand medical information. However, health literacy was not assessed using a validated instrument in the present study. Although participants reported their perceived understanding of medical information, this measure cannot be considered equivalent to objective health literacy. Therefore, the potential influence of health literacy on comprehension could not be directly evaluated. Previous research suggests that the relationship between health literacy and comprehension is complex and may interact with additional cognitive and contextual factors [1,21,22].

The IC process can also be influenced by additional factors, including language barriers and cultural differences, which may further affect the patient’s ability to understand information and make informed decisions [23,24,25,26].

The present study did not identify a significant association between comprehension and the willingness to sign an IC form. This finding suggests that decision-making in medical contexts is not determined solely by the level of understanding but is also influenced by emotional factors, such as anxiety, trust in the physician, and perceived risks [4,18,27,28].

Although previous studies indicate that education level may influence understanding, as individuals with lower levels of education may be less willing to ask for clarification, our results revealed no significant association between education and comprehension.

This discrepancy suggests that the quality of the material is the most important factor. When information is presented clearly, educational barriers are minimized. This shows that investing in plain-language, well-structured consent forms is more effective than assuming that higher education automatically leads to better understanding [29].

Similarly, no significant differences were observed between urban and rural participants, suggesting that the information was equally accessible across different demographic groups. This finding supports the idea that simplifying language and structure can reduce disparities in access to medical information.

Nevertheless, these findings should be interpreted with caution. The study sample was predominantly female, highly educated, and recruited primarily from urban areas through online convenience sampling. As a result, the observed comprehension levels may not fully reflect those of populations that are often considered more vulnerable during the IC process, including older adults, individuals with lower educational attainment, people with limited health literacy, and patients facing real illness-related stress.

The phenomenon of “therapeutic misconception,” whereby patients overestimate the benefits of medical interventions or participation in clinical trials, further illustrates the complexity of the IC process. This concept highlights the potential gap between understanding and expectations, which may influence both comprehension and decision-making [18,30,31,32,33].

Previous research has also shown that IC documents often exceed recommended readability levels, limiting patients’ ability to fully understand the information provided [34,35]. In this context, strategies such as the use of simplified materials, electronic formats, and interactive methods, including the “teach-back” technique, have been shown to improve comprehension [7,36,37]. Additionally, combining written, verbal, and multimedia information may further enhance understanding [38]. The presence of a trusted person during the consent process may offer emotional support, although this alone is insufficient in the absence of effective communication. Furthermore, adequate time allocated for the consent process is essential, as time constraints may reduce patients’ ability to process information effectively [18,39].

These findings reinforce the idea that the IC process should not be reduced to the mere signing of a document. Instead, it should be understood as a complex, dynamic, patient-centered process that involves effective communication and active patient participation, rather than a simple administrative formality [40].

Comprehension in real clinical settings may differ from that observed in a standardized questionnaire scenario, where emotional stress, illness burden, pain, anxiety and time constraints are less pronounced. In actual healthcare encounters, additional factors such as trust in the physician, fear related to the medical condition, and perceived dependence on the healthcare system may influence both understanding and decision-making. Therefore, caution is warranted when extrapolating these findings to real-world clinical consent situations.

### 4.4. Implications for Informed Consent Practice

Our study has important clinical implications. The discrepancy between self-perceived and objective comprehension suggests that health systems need to move toward proactive verification of understanding. Structured “teach-back” techniques may help verify patient understanding, particularly regarding aspects that are commonly misunderstood, such as the consequences of refusing a procedure. Such strategies can transform IC from a simple administrative formality to a genuine decision-making process [41].

Although the regression model was statistically significant, it explained a modest proportion of the variance in comprehension scores (*R^2^* = 0.16). This finding suggests that IC comprehension is influenced by multiple factors beyond those assessed in the present study. Variables such as objective health literacy, previous healthcare experiences, trust in healthcare professionals, cognitive characteristics, and contextual factors may also contribute to individual differences in understanding and should be explored in future research.

### 4.5. Limitations

This study has several limitations that should be acknowledged. First, the cross-sectional design does not allow causal relationships to be established, but only associations to be identified. Second, the use of an online questionnaire and a convenience sampling method may limit the generalizability of the findings and may have introduced selection bias. Response bias associated with self-reported measures and residual confounding due to unmeasured factors cannot be excluded. In addition, the study population was predominantly highly educated and from an urban environment, which may have influenced the overall level of comprehension. Although the questionnaire underwent expert review and pilot testing, formal validation was not performed. In addition, health literacy was not assessed using a validated instrument; therefore, its potential influence on comprehension could not be directly evaluated. An additional limitation is that no formal sample size calculation was conducted prior to data collection. Finally, the use of a hypothetical scenario may not fully reflect real-life clinical situations, where additional factors may influence both understanding and decision-making.

Future studies should evaluate IC comprehension in more diverse populations and real-world clinical environments, while also exploring interventions aimed at improving objective understanding.

## 5. Conclusions

In this study, participants achieved a mean comprehension score of 6.81 out of 8 when presented with a structured informed consent scenario. However, the findings should be interpreted in light of the study sample characteristics and the absence of validated cut-off values for the comprehension instrument. Our findings highlight a discrepancy between patients’ self-perceived comprehension and their objective comprehension, indicating that subjective confidence may not be a reliable indicator of actual understanding.

Furthermore, the lack of significant associations between formal education or professional background and comprehension scores suggests that understanding of the study scenario was relatively similar across these participant groups. However, because the present study did not directly compare different information materials or communication approaches, the factors underlying these findings remain uncertain.

These results reinforce the idea that IC should not be treated as a simple administrative formality or a signature on a page but as a dynamic, patient-centered communication process. Strategies aimed at improving patient understanding, including accessible information materials and effective clinician–patient communication, may support more informed decision-making. Further research in real clinical settings is needed to confirm the applicability of these findings across different patient populations and healthcare contexts.

## Figures and Tables

**Table 1:** Socio-demographic characteristics of participants (*N* = 275).

Variable	*n*	%
Sex		
Female	192	69.8
Male	83	30.2
Residence		
Urban	225	81.8
Rural	50	18.2
Education level		
Lower secondary	1	0.4
Upper secondary (High school)	73	26.5
Post-secondary	6	2.2
University	142	51.6
Postgraduate	53	19.3
Healthcare field		
Yes	23	8.4
No	252	91.6
Employment status		
Student	91	33.1
Employed	158	57.5
Self-employed	11	4
Unemployed	2	0.7
Retired	6	2.2
Other	7	2.5

*n* = number of participants; % = percentage.

**Table 2:** Multivariate linear regression analysis of factors associated with comprehension score.

Variable	*β*	SE	*p*-Value
Q19 (self-perceived comprehension)	0.381	0.115	<0.001
Healthcare field	−0.139	0.299	0.014
Education (university vs. non-university)	−0.027	0.182	0.627

*β* = standardized regression coefficient; SE = standard error.

## Data Availability

The study dataset contains participant-level information collected under ethics approval and informed consent procedures. For this reason, the dataset is not publicly deposited in an open repository. However, the data supporting the main findings of the study are available from the corresponding author upon reasonable request.

## Supplementary Materials

The following supporting information can be downloaded at: https://www.mdpi.com/article/10.3390/healthcare14121777/s1, Supplementary File S1: Questionnaire used in the study.

## Abbreviations

The following abbreviations are used in this manuscript:

IC	Informed consent
SD	Standard deviation
VIF	Variance inflation factor
