# Supplementary material for: Understanding Informed Consent: A Cross-Sectional Study of Objective and Self-Perceived Comprehension in Romania
Source: Healthcare (Basel). 2026 Jun 19;14(12):1777. doi: 10.3390/healthcare14121777 (PMC13299991; doi:10.3390/healthcare14121777)
Supplement: Supplementary file 1 [file healthcare-14-01777-s001.zip › healthcare-4336165-File S1-Questionnaire used in the study.pdf]

ASSESSMENT OF INFORMED CONSENT COMPREHENSION – ANONYMOUS SURVEY

INFORMED CONSENT FOR STUDY PARTICIPATION

You are invited to participate in a research study aimed at evaluating how information presented in informed consent forms is understood by the adult population.

Participation in this study is voluntary. The questionnaire is anonymous, and no personal data that could identify participants will be collected. You may stop completing the questionnaire at any time without any consequences. The collected data will be used exclusively for scientific purposes.

The study is conducted in accordance with the ethical principles outlined in the Declaration of Helsinki.

Completing the questionnaire takes approximately 6–8 minutes. No significant risks are anticipated. No financial benefits are offered; however, the results may contribute to improving medical communication and the informed consent process.

By continuing, you confirm that you are at least 18 years old, that you have read and understood the above information, and that you agree to participate in this study.

☐ I agree to participate voluntarily in this study

SECTION 1 – GENERAL INFORMATION AND MEDICAL EXPERIENCE

Q1. Age:
(numeric)

Q2. Sex:

- Male
- Female
- Prefer not to answer

Q3. Place of residence:

- Urban
- Rural

Q4. Education level:

- Lower secondary education
- Upper secondary education
- Post-secondary education
- University education
- Postgraduate education

Q5. Employment status:

- Student
- Employed
- Self-employed
- Unemployed
- Retired
- Other

Q6. Do you work in the healthcare field?

- Yes
- No

Q7. Have you previously signed an informed consent form for a medical procedure?

- Yes
- No
- Not sure

Q8. How often do you interact with the healthcare system (consultations, investigations)?

- Rarely (once a year or less)
- Occasionally (a few times per year)
- Frequently (monthly)
- Very frequently (multiple times per month)

SECTION 2 – HEALTH LITERACY

Q9. How well do you think you understand medical terms?
1 – Very poor
2 – Poor
3 – Moderate
4 – Good
5 – Very good

Q10. How would you rate the clarity of medical explanations you usually receive?
1 – Very unclear
2 – Unclear
3 – Acceptable

4 – Clear
5 – Very clear

SECTION 3 – STANDARDIZED INFORMED CONSENT SCENARIO

Imagine that your doctor recommends a minimally invasive diagnostic procedure. The procedure involves collecting a small biological sample or using a specialized medical instrument to obtain additional information about your health condition.

The potential benefits include establishing a more precise diagnosis and selecting an appropriate treatment. The procedure may be associated with certain risks, such as temporary local discomfort, mild pain, or, in rare cases, infection.

Your doctor will explain how the procedure is performed and the measures taken to minimize these risks. There are also alternatives, such as monitoring symptoms or using other non-invasive investigations, which can be discussed with your doctor.

Participation in the procedure is voluntary. You have the right to refuse the procedure or withdraw your consent at any time without affecting the quality of your medical care. Your medical data will remain confidential and will be used exclusively for medical purposes in accordance with data protection regulations.

By signing the informed consent, you confirm that you have read and understood the information and that you agree to undergo the procedure.

SECTION 4 – QUESTIONS ABOUT THE PRESENTED INFORMATION

Q11. According to the scenario, the main purpose of the procedure is to:

- Treat an existing condition
- Establish a more precise diagnosis
- Prevent the occurrence of a disease
- Contribute to scientific research

Q12. How are the risks of the procedure presented in the scenario?

- They are presented as nonexistent
- They are presented as minor and rare, such as discomfort or infection
- They are presented as severe and frequent, such as hemorrhage
- They are not mentioned

Q13. According to the scenario, establishing a more precise diagnosis aims to:

- Enable the selection of an appropriate treatment
- Immediately reduce symptoms
- Prevent other conditions

- Eliminate the need for future consultations

Q14. How are alternatives to the procedure presented?

- They are only indirectly suggested
- They are not mentioned
- They are clearly and explicitly presented
- They are presented as mandatory

Q15. According to the scenario, the patient has the right to:

- Accept the procedure only with the doctor's approval
- Refuse or withdraw consent at any time
- Refuse only before scheduling
- Transfer the decision to the doctor

Q16. According to the scenario, the patient's medical data:

- Are used for general scientific purposes
- Are publicly accessible
- Are used exclusively for medical purposes and kept confidential
- Are deleted immediately after the procedure

Q17. According to the information provided, what is the consequence of refusing the procedure?

- The patient loses access to treatment
- The doctor may refuse future consultations
- Medical care continues unchanged
- It is not mentioned

Q18. By signing the informed consent, the patient confirms that:

- They automatically accept all medical recommendations
- They have received and understood the information before agreeing
- There are no risks associated
- The responsibility lies entirely with the doctor

SECTION 5 – PERCEPTION OF INFORMATION

Q19. How well do you think you understood the information presented?
1 – Very poor
2 – Poor
3 – Moderate
4 – Good
5 – Very good

Q20. Would you need additional verbal explanations to make an informed decision?

- Yes
- No
- Not sure

Q21. Would you be willing to sign this informed consent based on the presented information?

- Yes
- No
- It depends

SECTION 6 – TEXT EVALUATION

Q22. How appropriate did you find the length of the text?
1 – Not appropriate at all
2 – Slightly appropriate
3 – Moderately appropriate
4 – Appropriate
5 – Very appropriate

Q23. How clear did you find the language used?
1 – Very unclear
2 – Unclear
3 – Acceptable
4 – Clear
5 – Very clear

SECTION 7 – VALIDATION QUESTION

To confirm that you have carefully read the questionnaire, please select "No" for this question.

- Yes
- No
